# Promoting positive emotions and instilling concern for the needs of others during the COVID-19 pandemic

**DOI:** 10.1371/journal.pone.0272922

**Published:** 2022-10-07

**Authors:** Belén Mesurado, María Cristina Richaud, Claudia E. Vanney, Concetta Pastorelli

**Affiliations:** 1 Instituto de Filosofía, Universidad Austral, Pilar, Argentina; 2 Consejo Nacional de Investigaciones Científicas y Técnicas (CONICET), Buenos Aires, Argentina; 3 Instituto de Ciencias de la Familia, Universidad Austral, Pilar, Argentina; 4 Sapienza Università di Roma, Roma, Italy; Chinese Academy of Sciences, CHINA

## Abstract

The purpose of this research is to study the efficacy of the home-based Hero program in promoting positive emotions and prosocial behavior during the COVID-19 pandemic. The sample included 237 12- to 15-year-old adolescents from Argentina. The level of positive emotions and prosocial behavior toward strangers, friends and family in the adolescent intervention group increased through the three evaluation periods. The Hero program was focused on recognizing one’s own emotions and provided an opportunity to reflect on different positive aspects of life, thus allowing a change in perspective related to immediate negative events. Moreover, the program provided an opportunity to change adolescents’ perspective from personal worries to concerns about others, including friends, family members, and even strangers in need.

## Introduction

The current coronavirus pandemic (COVID-19) has spread around the world, changing people’s lives. This pandemic is both a health crisis and a social and economic threat and has had a strong impact on children, adolescents and families. To prevent the spread of COVID-19, physical distancing policies, usually mandatory, have been developed, which has prevented the usual activities of social interaction. Such measures are likely to impact not only the economy and society but also people’s mental health and well-being.

In the particular case of Argentina, after the detection of the first case of COVID-19 on March 3, 2020, the national government decreed preventive and mandatory social isolation on March 20, and it lasted almost unchanged until November 29 of the same year, becoming the longest quarantine in the world, with serious social and economic consequences. The negative effects of physical and social distancing can be particularly dangerous for adolescents [1] since adolescence is a period in which social interaction is essential [2]. Adolescents are very sensitive to social stimuli and to the negative effects of social exclusion, and they can be particularly affected by social distancing, especially reduced contact with their peers [2].

Parents occupy a central position in the lives of children in early adolescence (ages 10–13), but this role is gradually taken over by friends at the end of early adolescence and at the beginning of middle adolescence (ages 14–17). In contrast to children of other ages, those in early and middle adolescence frequently spend more time with their friends than with their family while developing complex peer social relationships [3]. Indeed, trajectory studies found that parent–child warmth decreases from childhood to middle adolescence [4, 5], while the quality of close same-sex friendships improves at the transition from middle to late adolescence [6]. During early and middle adolescence, the influence of peers increases, and it becomes very important to obtain social approval [7–10]. Early and middle adolescence are affected by peer acceptance, rejection, and approval [11–13]. Moreover, adolescents’ perceptions of loneliness decrease from 12 to 18 years old [14], although both positive and negative attitudes toward being alone could be identified [15, 16]. Early and middle adolescence with low or moderately low loneliness in relation to parents and friends, combined with low or high positive and negative attitudes toward being alone, is related to an adaptive profile. In contrast, adolescents’ perceptions of a high level of loneliness in relation to parents or peers, or both combined with high positive and negative attitudes toward being alone, are related to a maladaptive profile [16].

Changes in the general social environment, such as compulsory physical distancing and reduced face-to-face social contact with peers, could have a significant effect on brain and behavioral development during adolescence [17]. Indeed, in a study with 415 adolescents conducted by researchers from Argentina [18], levels of depression, anxiety, and aggressiveness were found to be elevated. These results coincide with the results of a study performed by UNICEF on 8,444 adolescents and young people between the ages of 13 and 29 years in nine Latin American and Caribbean countries, in which 27% and 15% reported feeling anxiety and depression, respectively, in the last seven days, 46% reported being less motivated to do activities they normally enjoy, and 36% felt less motivated to do regular activities [19]. Moreover, a longitudinal study developed in middle adolescents, which included adolescents’ reports before and during COVID-19, showed increases in depression and isolation and decreases in friendship [20].

### Positive emotion

The COVID-19 pandemic shook the emotions of adolescents. During the pandemic, the deprivation of social contact and the increase in family conflict likely led to high levels of negative emotions in adolescents. Indeed, recent research has shown the negative impact that the COVID-19 pandemic has had on adolescents around the world [18, 21–23]. Recent studies have shown a significant increase in negative affect in early adolescence [24] and in middle adolescence [20] during the COVID pandemic compared to pre-COVID assessments. However, positive affect remained stable in early adolescence, and there was also a reduction in positive affect variability [24]. In contrast, the decreased positive affect in middle adolescence during the COVID pandemic [20] may be due to adolescents not having the opportunity to interact with outgroups and due to difficulties in social connectedness. Although there is more research on positive and negative affect in early and middle adolescents during the COVID-19 pandemic, there is relatively less research on positive emotions.

Fredrickson stated that positive emotions broaden us to new thoughts, activities and relationships and build lasting personal resources, which transform us into more resilient, gregarious, and healthy persons [25]. Fredrickson also stated that positive emotions are associated with “some personally meaningful circumstance (i.e., they have an object), are typically short-lived, and occupy the foreground of consciousness” p. 778 [25]. Positive emotions are those in which the valence of pleasure or well-being predominates and elicits adaptive behaviors that contribute to the achievement of personal and social goals [26–28]. Several positive emotions have been identified, but the most commonly studied are joy (general state of contentment, amusement and rejoicing), personal satisfaction (emotion related to the positive valuation of oneself), sympathy (tuning in to the emotion of others), serenity (state of peace and trust) and gratitude (feeling of appreciation to person or group of person for what they have done for you) [29]. Positive emotions can be analyzed individually or as part of a single construct called positive emotions [29].

Studies show that positive emotions may be a protective factor in times of uncertainty or crisis. Positive emotions can help to retrieve from posttraumatic or stressful events, such as terrorist attacks [30] or earthquakes [31]. Abundant evidence indicates that positive emotions reduce depressive symptoms, help overcome stress [30, 32] and counteract the undesirable effects of negative emotions [33]. Moreover, recent studies have shown that the presence of positive emotions during the COVID-19 pandemic was associated with a high level of resilience in adults [34]. The benefits of positive emotions may also be relevant to helping adolescents face the COVID-19 pandemic challenge. Consequently, it is highly desirable and necessary to promote positive emotions in adolescents during this time of uncertainty and potential conflict for adolescents.

### Prosociality

It is very common for people to be moved to donate money, time, material goods, and blood in different moments of their lives [35], especially during extreme situations [36]. In times of pandemic, prosociality is essential since it is necessary to help and support lonely people who are sick or elderly, collaborate by helping children complete their homework in a virtual manner, cooperate to complete home tasks, communicate with friends to help them with their homework or comfort them when they are sad.

Prosocial behavior is understood as a voluntary, intentional behavior that benefits another person and takes place without expecting any benefit in return [37–39]. Padilla-Walker and colleagues considered it essential to study prosocial behavior within the context of interpersonal relationships (2011, 2015) and stated that such behavior differs depending on whether it is directed at family, friends or strangers [40]. Padilla-Walker, Dyer [41] state that “prosocial behavior toward strangers is complex and may have very different motivations than prosocial behavior toward those with whom adolescents have a relationship” [p. 146].

Previous studies have shown that a large proportion of early and middle adolescents were involved in COVID-19 prosocial acts toward strangers (e.g., grocery shopping for people at risk, donations food, money or house household supplies), friends (e.g., giving a gift to a friend because he or she was quarantined) and family (e.g., help with household chore) [36]. However, prosocial behaviors among adolescents presented variations depending on the target or level of need of the person during the pandemic. Adolescents who participated in the Dictator game, in which they had to split 10 coins with a second person, gave away more resources to those who were in need or friends than unfamiliar others. In fact, adolescents were willing to share 78% of their resources with a doctor in the hospital, 69% of their resources with people with COVID-19, 63% with people with a poor immune system, 51% of their resources with a friend, and only 39% with unfamiliar others [42].

Moreover, longitudinal studies including a pre-COVID and a during COVID evaluation in adolescents found that there was no change in prosocial behavior toward unfamiliar others and society at large; however, there was a decrease in prosocial behavior toward familiar people, such as friends [42]. Another longitudinal study, which included four evaluation times during COVID-19, showed that there was a decrease in prosocial behavior toward COVID-19 targets (doctors, people with COVID-19, and people with a poor immune system), while prosocial behavior toward familiar others (e.g., friends) and unknown others remained stable across four evaluation times [43].

Taking care of and supporting others is stimulating and empowering: there is considerable evidence that helping others increases the mental health of the caring and supportive “giver” [44]. Additionally, the presence of prosocial behavior during the COVID-19 crisis among adolescents can promote positive psychological functioning. Recent studies have shown that people who donate money to charities (e.g., institutions that recollected elements to protect health workers again from COVID-19 or food for children unable to attend school due to COVID-19) reported higher levels of meaningfulness, empathy, social connectedness, positive impact and positive affect than people who spend money for themselves [45]. It is important to highlight that prosocial behaviors are also associated with positive emotions. Indeed, some authors postulate that there is a positive feedback loop between some prosocial behavior (e.g., spending money for others or donating money) and positive emotions [46]. In other words, prosocial behaviors may promote positive emotions, and in turn, positive emotions can promote helping others.

### Hero program

Positive technology, a new paradigm aimed at using technology to improve human and society flourishing, has achieved unprecedented growth in recent decades. Based on this paradigm, Hero was designed to promote positive emotions and prosocial behavior toward family, friends, and strangers in adolescents. This promotion is made in a direct and indirect way through five related variables: empathy, emotional recognition, positive emotions, gratitude, and forgiveness [47]. Hero is a self-administered online program that can be completed from home, taking advantage of the fact that being digitized is very friendly for adolescents since young people are among those who use the most digital communication technology [48].

In times of pandemic when individuals must stay home because social isolation is necessary, a program with these characteristics is ideal because it can be implemented without having to leave home. The Hero program, which is the first such program to be developed online, includes five modules, represented by five islands, each of which lasts approximately 45 minutes and promotes one of the five socioemotional variables mentioned above. During administration of the program, the participant is guided by a "Sensei" who helps him or her in the different activities or games that he or she must complete [47, 49]. The role of the Sensei into the program is to present the different activities and give activities instructions to adolescents.

As the program progresses, the participant accumulates points since each activity has an assigned value. At the beginning of the program, the avatar that represents the teenager boards a boat that travels to the different islands. The islands, which appear in the sequence of empathy, gratitude, positive emotions, forgiveness and prosocial behavior, can be visited only once per session, and the sequence of islands or activities cannot be changed [47]. Each intervention session consisted of a psychoeducational video about the socioemotional variable to be worked on and two or three activities aimed at promoting it. For example, the first session includes a video about four adolescents who have an interpersonal problem, and the negative event is solved by exercising empathy. During the video, adolescents receive information about the importance of exercising empathy and suggestions about how to exercise it in daily situations. A similar procedure is made in the other videos related to gratitude, positive emotions, forgiveness and prosocial behavior. The following are some of the activities included in the Hero program. In the empathy session, the program trains adolescents in emotional recognition exercises using previously validated photos of children. The task is to identify the emotion expressed by the photo. In the gratitude session, for example, the book of gratitude, in which the adolescents have the opportunity to recall and write about situation or events for which they are grateful. This strategy to promote gratitude was used in previous studies [50]. The positive emotions session includes relaxation exercises using landscape images and relaxing music. The forgiveness session includes activities aimed at reflecting on situations in which the adolescent made a mistake and was forgiven by another person. Subsequently, the adolescent is asked to think about situations in which another person offends him or her and he or she has the opportunity to exercise forgiveness by expressing it in a letter. Finally, the prosocial behavior session aims to make teenagers aware of different social initiatives carried out by people or organizations that help people in need and with which they can get involved. A deeper description of the activities included in each island can be found in Mesurado, Oñate [51].

This program has proven its effectiveness in Argentina and other Latin American countries [51]. Indeed, with respect to Argentina, the efficacy of the program was analyzed in three evaluation periods (preintervention, postintervention and maintenance), and the Hero program increased prosocial behavior toward strangers, friends, and family members among the participants with respect to the control group. In addition, the program was effective in both women and men.

Based on this background, the purpose of this research is to study the efficacy of the home-based Hero program in promoting positive emotions and prosocial behavior among adolescents during the COVID-19 pandemic.

We hypothesize that

1. Adolescents who participate in the home-based program Hero will develop higher levels of positive emotions than a normative group of adolescents (control group). In the case of the control group, positive emotions will decrease.
2. Adolescents who participate in the home-based program Hero will develop higher levels of prosocial behavior toward strangers, friends and family than the normative group of adolescents. In the case of the control group, prosocial behavior will remain stable or decrease.

## Materials and methods

The study and procedures were approved by the Institutional Review Board at Universidad Austral [CIE 20–058].

### Research design

Six schools were interested in participating in the Hero program. We used a cluster-randomized trial design with a pretest, posttest, and 3-month follow-up. The students of three schools were included in the control group waiting list, while the students of the other three schools were included in the intervention group. The inclusion and exclusion criteria were 1. Aged between 12 and 15 years old, 2. Access to a computer and internet connection at home, 3. Not participating in another intervention program, and 4. There are no reports of internalizing or externalizing behavior disorders. This research began in April 2020, three weeks after mandatory confinement for COVID-19 started in Argentina, and the follow-up measure was conducted in August 2020. During the course of the investigation, the Argentine Ministry of Health reported 408,426 positive cases of COVID-19 and 8,498 deaths [52]. Of the total positive cases, 49.1% were women and 50.9% were men. The main age groups affected in the registered cases correspond to people between 20 and 59 years old, with an average age of 36 years. On March 19, 2020, a nationwide lockdown was established in Argentina [53]. The lockdown included the suspension of face-to-face classes throughout the Argentine academic year that began in March and ended in December of each year. Likewise, the confinement included restrictions on social gatherings and the closure of public places, and access to public transport was reserved for only essential personnel (doctors, police, nurses, etc.). The lockdown was partially lifted on November 8, 2020 [54].

### Participants

**Fig 1:**
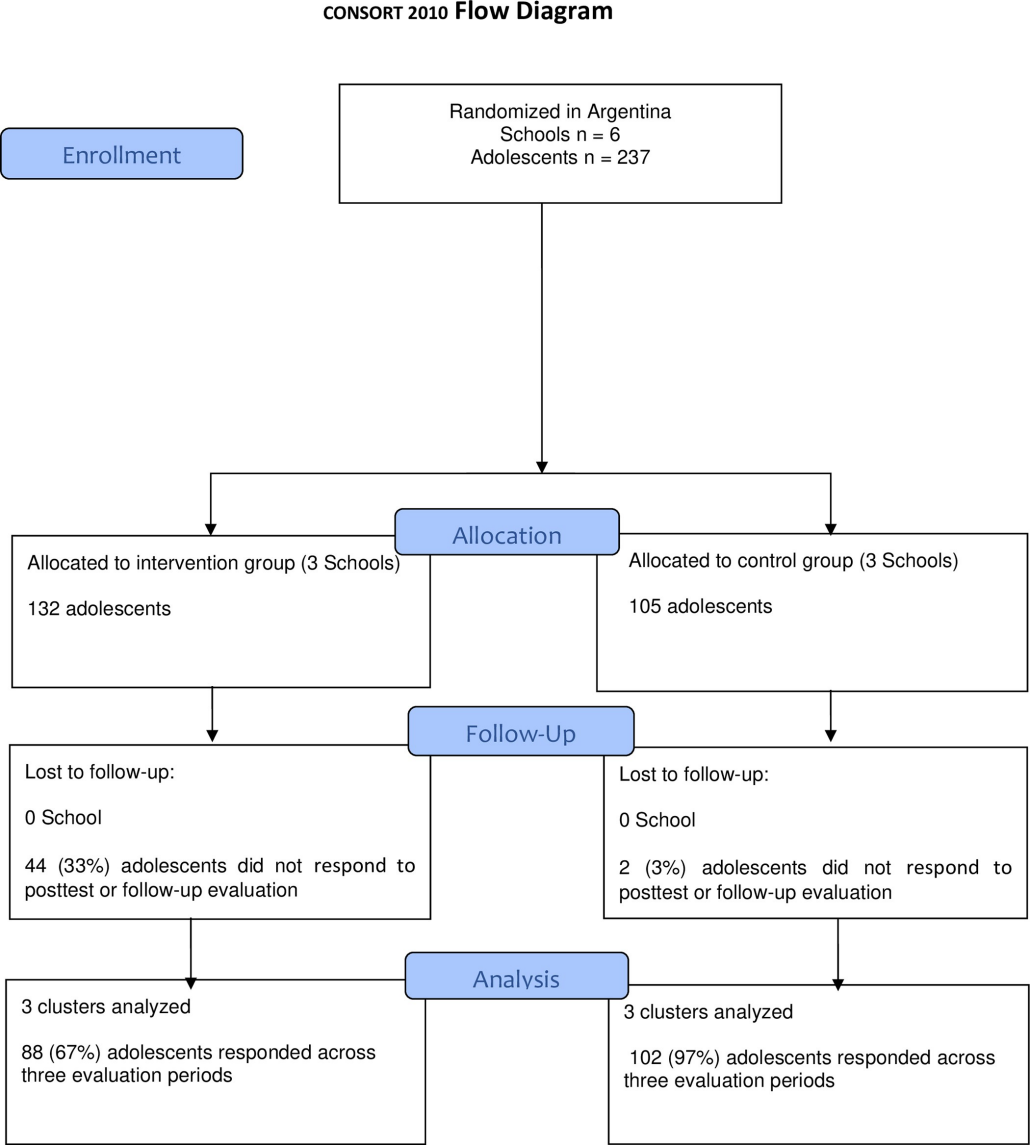
CONSORT flow diagram.

We followed the Consolidated Standards of Reporting Trials (CONSORT) recommendations to develop the flow diagram shown in Fig 1 [55, 56]. Six institutions were randomly included in the experimental or control group. The institutions included in the control group were offered the possibility of participating in the Hero program after the end of the research. The institutions had similar characteristics, all of which were private educative intuitions located in Buenos Aires, Argentina. These schools offer kindergarten, primary education, and secondary education. In the case of the secondary level, students have a double school day. Each institution has approximately 400 students at the secondary level.

Initially, the sample included 237, from grade 1 to 4, aged 12 to 15 years from Buenos Aires, Argentina. Thirty-three percent of the intervention group did not complete the posttest or follow-up evaluation, while three percent of the control group did not. Finally, 88 adolescents completed the three evaluation times: 51% females in the intervention group (M age = 13.52, SD = 1.04), and 102 adolescents completed the three evaluation times, 58% females, in the waiting list control group (M age = 13.59, SD = .91). There were no differences between the experimental and control groups regarding age [F(1, 232) = .31, p = .57] and gender [χ^2^ (1) = .99, p = .32].

The adolescents reported that 56% of the mothers had completed university studies, 27.4% completed secondary school, 11.5% completed elementary school, and 5.1% said they did not know the highest level of education attained by their mother. On the other hand, the adolescents who participated in the study reported that 47% of their fathers had completed university studies, 29% completed secondary school, 13% completed elementary school and 11% said they did not know the highest level of education attained by their father. No differences were found related to the father’s and mother’s educational levels between schools included in the study.

The participation of the adolescents was voluntary and anonymous; adolescents used a pseudonym within the online program. Moreover, participants did not receive any type of compensation in return.

### Instruments

The Positive Emotions Questionnaire developed by Oros [57] was used to measure adolescents’ positive emotions. The instrument includes 23 items that assess different types of positive emotions: joy (e.g., “I am almost always happy”), sympathy (e.g., “If I see a child cry, it makes me want to cry too”), gratitude (e.g., “I value when others help me”), serenity (e.g., “Even if I have problems, I still stay calm”), and personal satisfaction (e.g., “I feel such as I am very valuable”). Oros [57] suggests the possibility of using the average of all items as a general measure of positive emotion. The adolescent is asked to indicate his or her degree of agreement with the statements contained in the questionnaire using the following answer options: 3 = yes, 2 = somewhat, or 1 = no.

The Spanish version of the Prosocial Behavior Toward Different Targets Scale [58] was used to measure adolescents’ prosocial behavior. The original scale was developed by Padilla-Walker and Christensen [40] based on the Kindness and Generosity subscale of The Values in Action Inventory of Strengths [59]. The scale includes 27 items that assess three types of prosocial behavior: toward strangers (e.g., “I watch out for others, even if I do not know them”), toward friends (e.g., “I watch out for my friends”), and toward family members (e.g., “I watch out for members of my family”). The adolescent is asked to indicate the degree to which they are described by the statements included on the scale using a five-point scale from 5 (“very much such as me”) to 1 (“not such as me at all”).

### Procedure

Austral University offered a presentation of the Hero program to directors of educational institutions in Argentina through the Zoom platform. In that meeting, the general characteristics of the program, the theoretical background of its development and the intervention procedure proposed for the implementation of the program were presented. After the introductory meeting, directors interested in participating in the research were asked to contact the project director: six institutions were interested in implementing the program. Subsequently, a second meeting was held with the directors of each institution and the teachers who would coordinate the intervention in their educational institution. In this meeting, personalized training was provided in which the theoretical foundation of the Hero program and its activities were shown. Moreover, they were shown, in detail, the activities that constitute the program, the reward system in the scores, and the instruments used to measure positive emotions and prosocial behavior. They were also shown how the Hero program collects and protects anonymized data that is later used for analysis of the program’s effectiveness. Finally, the implementation was coordinated, for example, the date and time at which each group of students would participate through virtual platforms. At the end of the meeting, each director and teacher received a brief document with all the topics presented at the meeting and a program user’s manual. The teacher training lasted two and a half hours. Then, the directors and teachers could try the program at their homes and play with the program as their students would later.

The directors of the schools were in charge of managing the informed consent of the adolescents’ parents and obtaining the assent of the students to participate in the program. Once these authorizations were obtained, weekly synchronous meetings were coordinated through Google Meet with a group of approximately 20 adolescents per session, a teacher from the educational institution, and a psychologist trained in the use of the Hero program. According to the country’s regulations related to the protection of minors, educational institutions require the presence of at least two adults when interacting with students in virtual environments. For this reason, during the virtual intervention sessions, in addition to the psychologists directing the program, a teacher in charge of the course was also present. The role of the psychologist is introducing the activity planned to be carried out that day and addressing any doubts or technical problems that the adolescents might have regarding the use of the program. The weekly meetings lasted between 45 minutes and one hour each. In the first meeting, the adolescents were asked to generate a user within the program and to complete the pretest evaluation. The following week, the adolescents began the empathy, gratitude, positive emotions, forgiveness and prosociality intervention sessions. At the seventh meeting, participants completed the posttest assessment, and at the eighth meeting (12 weeks later), they completed the follow-up assessment. In the case of the participants included in the waiting list control group, they participated only in the pretest, posttest, and follow-up evaluations following the same timeline as the experimental group. After the evaluations, they continued with their usual school activities.

### Data analysis approach

We used latent growth curve models (LGCMs) to evaluate the effect of the Hero intervention program in promoting prosocial behavior and positive emotions. LGCMs are appropriate to identify how a variable changes over time, and they are used when a researcher has a hypothesis about how the variable changes over time [60]. In our case, we expect to find no change or a decrease in the prosociality and positive emotions of adolescents in the control group during the pandemic, while we expect to find an increase in those variables in participants in the intervention group.

In the case that LGCMs are used to test the efficacy of the intervention program, the intercept and the slope are identified as growth latent factors using the mean of the pretest, posttest, and follow-up of the variables (in our study, positive emotions and prosociality). Moreover, the intercept and the slope have a direct effect on the pretest, posttest, and follow-up measures. Furthermore, the factor loadings can be fixed a priori. The intercept is considered the "initial status" of the variable, while the slope represents the “change over time”.

We follow three steps in the development of the analysis of each of our variables (positive emotions and prosocial behavior toward strangers, friends and family):

Step 1. Study of the development of positive emotions and prosociality for adolescents who did not participate in the intervention (control group) to identify the normative change in the variables.
Step 2. Study of the development of positive emotions and prosociality of adolescents who participated in the intervention to assess the changes in variables promoted by the program.

To develop steps 1 and 2, a series of LGCMs was tested in the following sequence. First, a no-change model that assumes no change in the variables across pretest, posttest and follow-up measures was studied. Second, a linear change model that assumes linear growth between time points was considered. Because the intervention lasted 5 weeks and the follow-up measurement was performed 12 weeks after the intervention had ended, the loadings on the slope factor were fixed at 0, 1, and 3. Third, a nonlinear change model was tested; in this case, the slope factor loadings of the pretest and follow-up were fixed at 0 and 1, respectively, and the slope factor loading of the posttest was freely estimated. Comparison of the chi-squared values was used to select the best-fitting model and to determine the growth curve. We compared a series of nested models for each of our variables. Initially, we compared the chi-squared values of the model of no change vs. linear change; when this difference was statistically significant, it indicated that the linear change model had a better fit. In this case, the next step was to analyze the chi-squared difference between the linear change model and the nonlinear change model. On the other hand, in the event that the difference between the nonchange model vs. the linear change was not significant, the nonchange model vs. the nonlinear change model was analyzed. A statistically significant difference in this case indicated that the nonlinear change model had a better fit than the no change model.

We also used the comparative fit index (CFI) and Tucker–Lewis index (TLI) as model fit indices and the standardized root mean square residual (SRMR) as an error index. According to Wang and Wang [61], the cutoff for CFI and TLI is.90; however, Hu and Bentler [62] suggest.95. Moreover, an SRMR less than.08 indicates a well-fitting model; however, the evaluation of the other adjustment indices should not be omitted [63].

Step 3. Once the form of the growth curve was determined, we used the total sample to study the effect of the Hero intervention program on the intercept and slope of positive emotions and prosociality (toward strangers, friends and family) separately in four models. Furthermore, the models were estimated with gender and age as time-invariant predictors. The chi-squared, CFI, TLI, and SRMR were used to analyze the model fit.

All latent growth curve models were carried out using MPLUS 8.5, including the 237 participants [64]. In all models studied, the missing at random method (MAR) was implemented to impute the missing data. The MAR method assumes that dropout at posttest or follow-up could be related to the scores from a previous assessment (pretest or posttest) [65].

## Results

### Preliminary analyses

Initially, it was analyzed whether there were statistically significant differences between adolescents who completed the program and those who dropped out in baseline (pretest) levels of positive emotions and prosocial behavior toward strangers, friends and family. The results indicated that they had similar initial levels of positive emotions [F(1, 232) = 2.11, p = .15], prosocial behavior toward strangers [F(1, 232) = .65, p = .42], prosocial behavior toward friends [F(1, 232) = 1.44, p = .23] and prosocial behavior toward family members [F(1, 232) = .90, p = .34]. Additionally, no demographic differences were found [gender χ^2^ (1) = .29, p = .59, and age F(1, 232) = .29, p = .58].

Table 1 displays the means and standard deviations of the variables included in this study for the total sample and males and females separately.

**Table 1:** Mean and standard deviation of the variables.

	Pretest			Posttest			Follow-up		
	Total sample M(SD)	Male M(SD)	Female M(SD)	Total sample M(SD)	Male M(SD)	Female M(SD)	Total sample M(SD)	Male M(SD)	Female M(SD)
PB_Strangers									
Control group	3.14 (.67)	2.99 (.66)	3.27 (.65)	3.13 (.74)	2.99 (.69)	3.26 (.77)	3.16 (.77)	2.99 (.84)	3.33 (.65)
Intervention group	3.15 (.51)	2.99 (.69)	3.25 (.70)	3.51 (.51)	3.32 (.73)	3.65 (.67)	3.61 (.60)	3.37 (.76)	3.79 (.75)
PB_Friends									
Control group	4.36 (.57)	4.14 (.62)	4.57 (.42)	4.24 (.61)	3.96 (.66)	4.48 (.45)	4.27 (.62)	4.08 (.63)	4.46 (.56)
Intervention group	4.32 (.46)	4.12 (.80)	4.48 (.52)	4.43 (.34)	4.28 (.59)	4.55 (.55)	4.35 (.48)	4.07 (.66)	4.55 (.65)
PB_Family									
Control group	4.02 (.76)	4.04 (.70)	4.01 (.79)	4.00 (.79)	4.00 (.80)	4.00 (.80)	3.98 (.78)	3.97 (.80)	3.99 (.78)
Intervention group	3.89 (.59)	3.77 (.76)	3.40 (.77)	4.16 (.40)	4.00 (.60)	4.28 (.63)	4.12 (.52)	3.90 (.71)	4.28 (.70)
PE									
Control group	2.38 (.31)	2.41 (.27)	2.36 (.34)	2.39 (.33)	2.40 (.28)	2.39 (.36)	2.40 (.32)	2.43 (.28)	2.37 (.35)
Intervention group	2.49 (.08)	2.46 (.28)	2.52 (.30)	2.60 (.09)	2.59 (.27)	2.60 (.34)	2.59 (.10)	2.57 (.29)	2.61 (.34)

Note: Means and standard deviations (in parentheses), PB_strangers: prosocial behavior toward strangers, PB_friends: prosocial behavior toward friends, PB_Family: prosocial behavior toward family, PE: positive emotions

### Positive emotions

**Fig 2:**
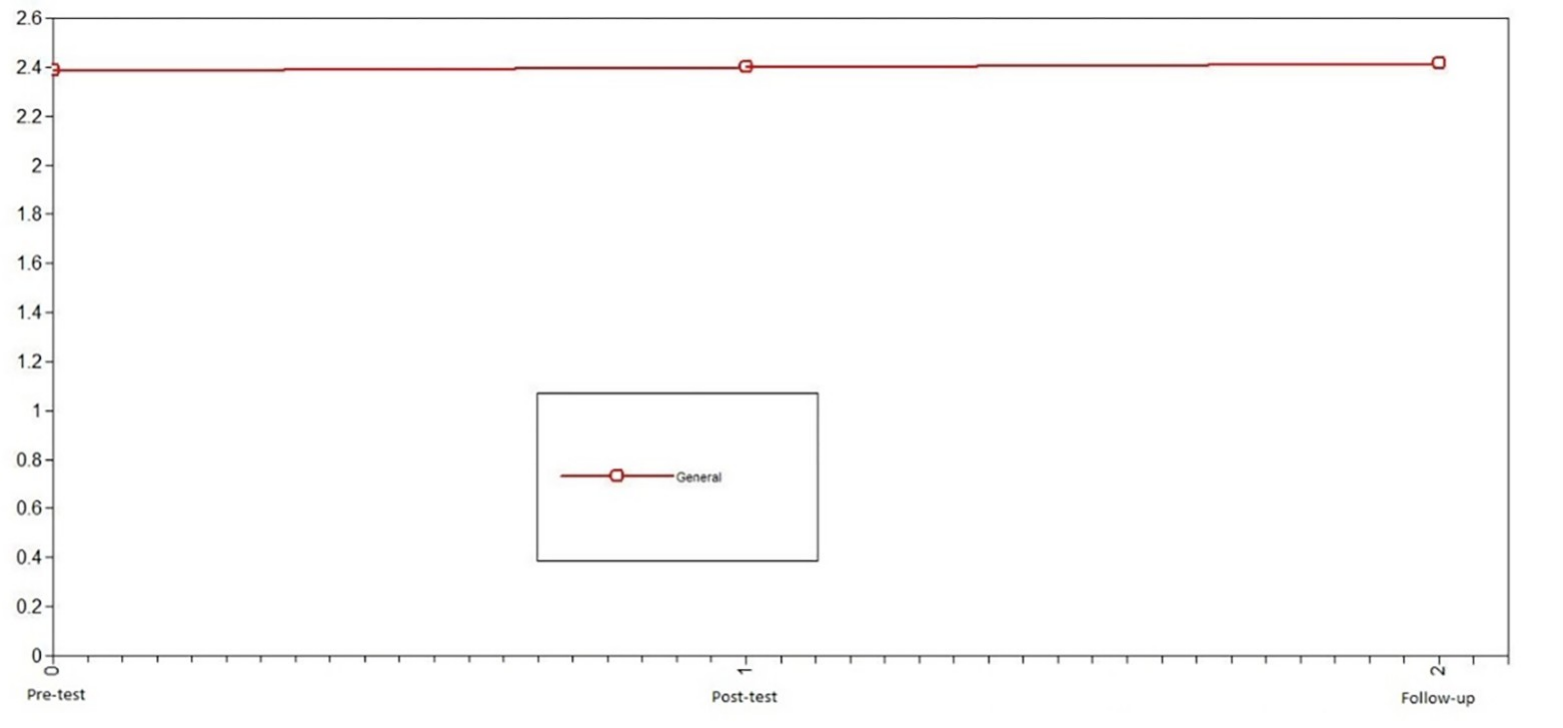
Latent growth curve models of positive emotions of the control group. The best fit in the control group was the no change model.

First, we studied the development of positive emotions in adolescents who did not participate in the intervention (control group) to identify the normative change in the variable. Using LGCMs, we first compared the chi-squared of the no change model vs. linear change model for positive emotions. Because this difference was not significant, we compared the no change model vs. nonlinear change model; this comparison was also not significant. Consequently, the model that presented the best fit in the control group was the no change model X^2^ = 3.91, df = 6, CFI = 1, TLI = 1, SRMR = .10 (see comparison model in Table 2). Notably, the level of error of the model fit is slightly high. For the control group, positive emotions were stable across the three evaluation times (see Fig 2).

**Table 2:** Fit indices and parameter estimates for the control and intervention groups.

		Fit Indices								LC parameters				
	Model	X^2^	df	CFI	TLI	SRMR	MC	X^2^	df	I mean	I Var	S Mean	S Var	I-S Cov
PB_Strangers Control	**A. No change**	**8.68**	**6**	**.99**	**.99**	**.07**				**3.10** ***	**.36** ***	-	-	-
	B. Linear change	3.73	3	.99	.99	.05	A vs. B	4.95	3					
	C. Nonlinear change	.58	2	1	1	.03	A vs. C	8.10	4					
PB_Strangers Intervention	A. No change	41.65***	6	.86	.93	.26								
	B. Linear change	15.76***	2	.95	.92	.04	A vs. B	25.89***	4					
	**C. Nonlinear change**	**.02**	**1**	**1**	**1**	**.01**	**B vs. C**	**15.74** ***	**5**	**3.15** *	**.35** **	**.46** ***	**.32** **	**-.13**
PB_Friend Control	A. No change	42.36***	5	.87	.92	.41								
	**B. Linear change**	**8.71** **	**2**	**.98**	**.97**	**.02**	**A vs. B**	**33.65** ***	**3**	**4.39** ***	**.26** ***	**-.05** ***	**.02** ***	**-.01**
	C. Nonlinear change	7.44**	1	.98	.93	.02	B vs. C	1.27	4	4.39***	.26***	-.16***	.20***	-.03
PB_Friend Intervention	A. No change	25.02***	6	.77	.89	.33								
	B. Linear change	8.91	2	.92	.88	.36	A vs. B	16.11***	4	4.37***	.21***	-.01	.05***	-.01
	**C. Nonlinear change**	**1.78**	**1**	**.99**	**.97**	**.01**	**B vs. C**	**7.13** ***	**5**	**4.33** ***	**.40** ***	**.09** *	**.29** ***	**-.21** ***
PB_Family Control	A. No change	23.02	5	.96	.98	.19								
	**B. Linear change**	**.97**	**2**	**1**	**1**	**.02**	**A vs. B**	**22.05** ***	**4**	**4.04** ***	**.52** ***	**-.03** *	**.04** ***	**-.03** *
	C. Nonlinear change	.56	1	1	1	.01	B vs. C	.41	5					
PB_Family Intervention	A. No change	35.24***	6	.73	.87	.36								
	B. Linear change	16.96***	2	.86	.79	.06	A vs. B	18.28***	4					
	**C. Nonlinear change**	**.24**	**1**	**1**	**1**	**.02**	**B vs. C**	**16.72** **	**5**	**3.90** ***	**.52** ***	**.24** ***	**.24** ***	**-.21** ***
PE Control	**A. No change**	**3.91** ***	**6**	**1**	**1**	**.10**				**2.40** ***	**.08** ***	-	-	-
	B. Linear change	.99***	2	1	1	.06	A vs. B	2,92	4					
	C. Nonlinear change	.18	1	1	1	.04	A vs. C	3.73	5					
PE Intervention	A. No change	30.22***	5	.84	.90	.16								
	B. Linear change	12.82***	2	.93	.90	.13	A vs. B	17.40***	3					
	**C. Nonlinear change**	**.66**	**1**	**1**	**1**	**.09**	**B vs. C**	**12.16** **	**4**	**2.50** ***	**.06** ***	**.10** ***	**.01**	**.01**

Note: The model that presented the best fit is highlighted

*** *p* < .001

** *p* < .01

*p < .05; PB_strangers: prosocial behavior toward strangers, PB_friends: prosocial behavior toward friends, PB_Family: prosocial behavior toward family, PE: positive emotions

**Fig 3:**
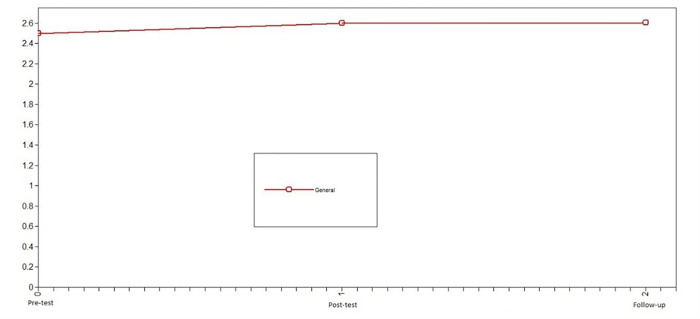
Latent growth curve models of positive emotions of the intervention group. The model that presented the best fit was the nonlinear change model.

Subsequently, we studied the development of positive emotions in adolescents who participated in the intervention to assess the change in the variables promoted by the Hero program. In this case, the developmental trajectory of positive emotions differed over time. The model that presented the best fit was the nonlinear change model: X^2^ = 0.66, df = 1, CFI = 1, TLI = 1, SRMR = .09 (see Table 2). Notably, the level of error of the model fit is slightly high. For the intervention group, positive emotions increased from pretest to posttest and remained stable from posttest to follow-up (see Fig 3).

In both the control and intervention groups, we found a significant intercept mean, which indicates individual differences in initial (pretest) positive emotions scores. Moreover, the variance of the intercept was also significant, which indicates large individual differences in the pretest.

We also found a significant slope mean, which indicates that, on average, in the intervention group, there was a nonlinear increase in latent positive emotions scores over time (see Tables 1 and 2). In addition, because the variance of the slope was not significant, we can assume that adolescents’ growth curves in the intervention group did not differ in terms of slope. No correlation was observed between the intercept and slope.

**Fig 4:**
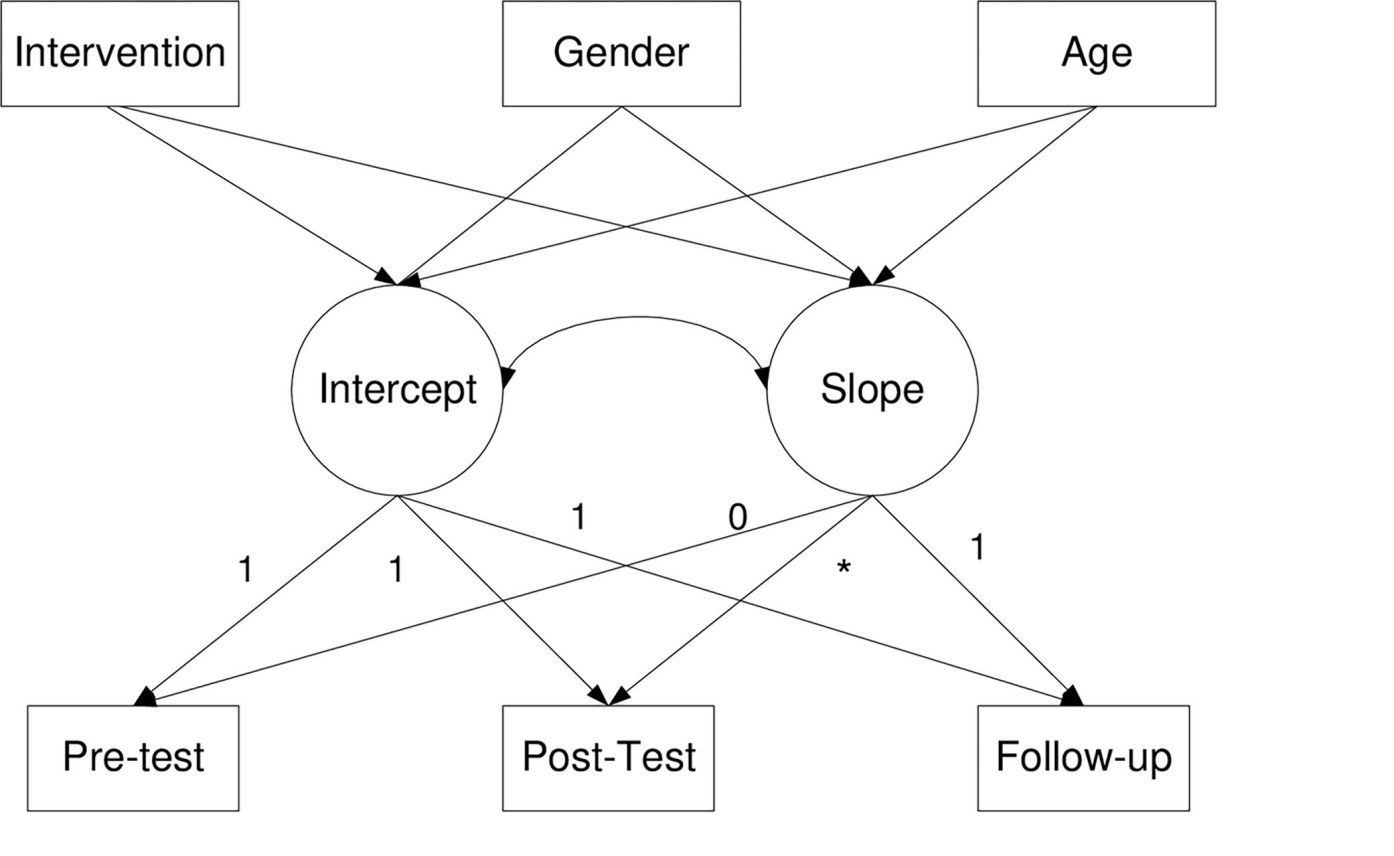
The effect of the Hero intervention program on the intercept and slope of positive emotions and prosociality (toward strangers, friends and family) controlling for age and gender.

Finally, we used the total sample to test the model displayed in Fig 4. We studied the effect of the intervention, gender, and age on the intercept and slope of positive emotions. The model showed a good fit: X^2^ = 1.01, df = 4, p = .91, CFI = 1, TLI = 1, SRMR = .04. The effect of the Hero program on positive emotion growth was significant (*β* = .09, *SE* = .01, *p =* .05), while the effects of gender and age were not significant (see Table 3). The R^2^ for the pretest was 72% (*p≤*. 001), that for the posttest was 80% (*p≤*. 001) and that for the follow-up was 77% (*p≤*. 001).

**Table 3:** Latent growth curve models.

	Model 1 PB_strangers β (SD)	Model 2 PB_friend β (SD)	Model 3 PB_family β (SD)	Model 4 PE β (SD)
Correlations				
Slope-Intercept	.45 (.85)	.18 (.29)	-.01 (.35)	.01 (.01)
Standardized beta weights				
Intervention → Intercept	-.07 (.09)	-.09 (.10)	-.08 (.08)	.20 (.08)
Gender →Intercept	-.02 (.09)	.10 (.10)	.06 (.08)	.13 (.08)
Age →Intercept	.21 (.09)*	.16 (.10)	-.06 (.08)	.05 (.08)
Intervention →Slope	.73 (.24)**	.36 (.18)*	.54 (.21)**	.09 (.03)*
Gender →Slope	.25 (.15)	.08 (.13)	.03 (.15)	-.01 (.03)
Age →Slope	-.34 (.18)	-.10 (.13)	-.15 (.18)	-.01 (.01)
Intercept →Pretest	.74 (.06)***	.68 (.06)***	.81 (.04)***	.85 (.03)
Intercept →Post test	.69 (.07)***	.70 (.08)***	.86 (.07)***	.78 (.05)
Intercept →Follow-up	.63 (.08)***	.65 (.06)***	.79 (.07)***	.77 (.06)
Slope →Pretest	-	-	-	-
Slope →Post test	.39 (.12)***	.55 (.18)***	.35 (.12)**	.24 (.12)*
Slope →Follow-up	.41 (.15)**	.24 (.13)*	.27 (.14)*	.25 (.14)

Note

*** *p* < .001

** *p* < .01

*p < .05; PB_strangers: prosocial behavior toward strangers, PB_friends: prosocial behavior toward friends, PB_Family: prosocial behavior toward family, PE: positive emotions

### Prosocial behavior toward strangers

**Fig 5:**
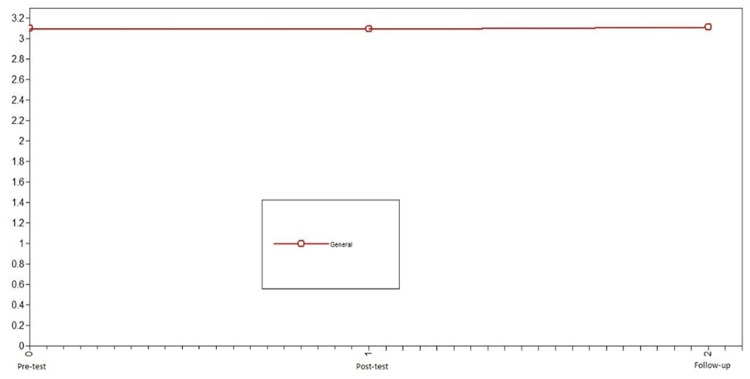
Latent growth curve models of prosocial behavior toward strangers of the control group. The best fit in the control group was the no change model.

First, we studied the change in prosocial behavior toward strangers in adolescents who did not participate in the intervention (control group) to identify the normative change in the variable. Using LGCMs, we first compared the chi-squared of the no change model vs. linear change model for prosocial behavior toward strangers. Because this difference was not significant, the no change model vs. nonlinear change model was compared. This comparison was also not significant. Consequently, the model that presented the best fit in the control group was the no change model: X^2^ = 8.68, df = 6, CFI = .99, TLI = .99, SRMR = .07 (see comparison in Table 2). For the control group, prosocial behavior toward strangers was stable across the three evaluation times (see Fig 5).

**Fig 6:**
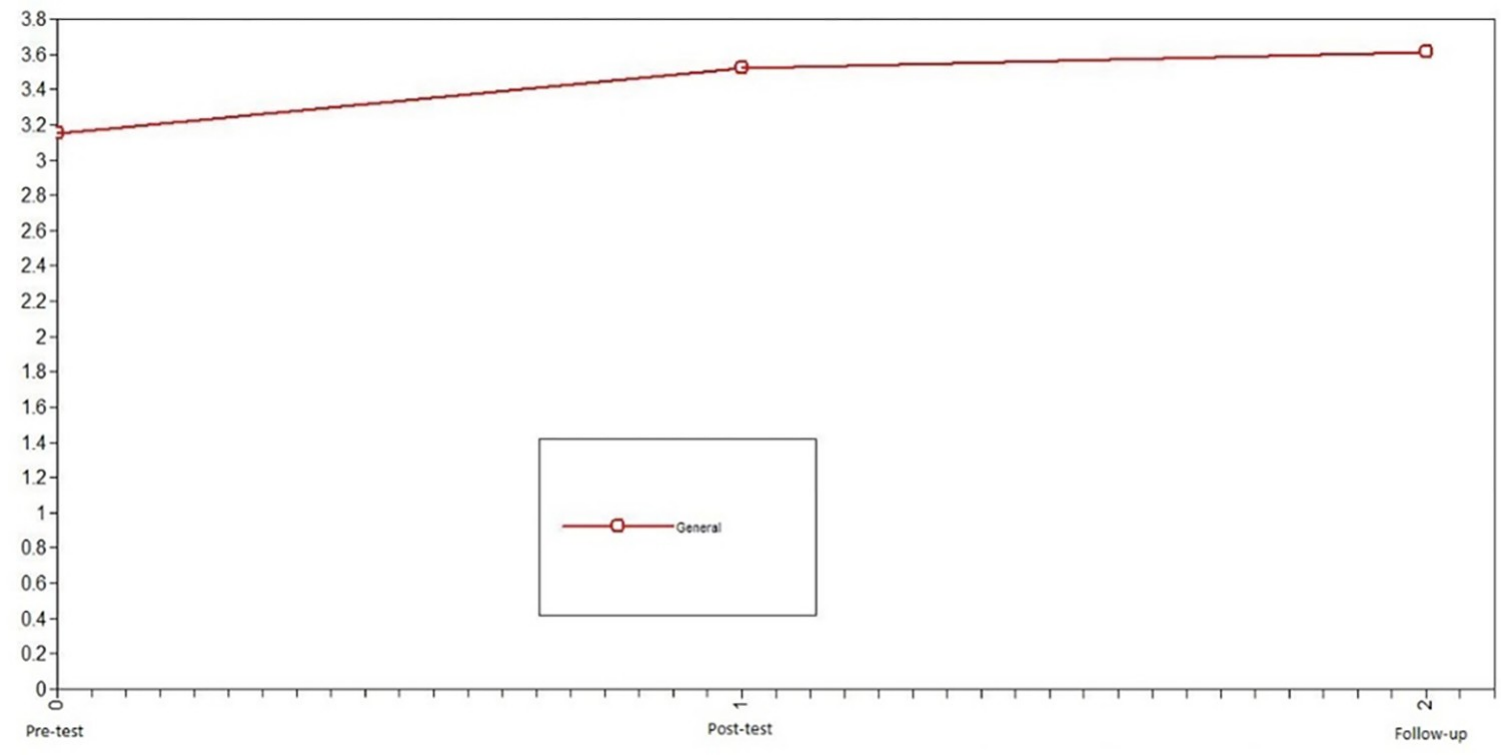
Latent growth curve models of prosocial behavior toward strangers of the intervention group. The model that presented the best fit was the nonlinear change model.

Subsequently, we studied the development of prosociality toward strangers in adolescents who participated in the intervention to assess the change in variables promoted by the Hero program. In this case, the developmental trajectory of prosocial behavior toward strangers in the intervention group differed over time. The model that presented the best fit was the nonlinear change model: X^2^ = 8.68, df = 1, CFI = .99, TLI = .99, SRMR = .07 (see Table 2). For the intervention group, prosocial behavior toward strangers increased from pretest to posttest and remained stable from posttest to follow-up (see Fig 6).

In both the control and the intervention groups, we found a significant intercept mean, which indicates individual differences in initial (pretest) prosocial behavior toward strangers. Moreover, the variance of the intercept was also significant, which indicates large individual differences in the pretest.

Additionally, we found a significant slope mean, which indicates that, on average, in the intervention group, there is a nonlinear increase in latent prosocial behavior toward strangers over time (see Tables 1 and 2). In addition, because the variance of the slope is significant, we can assume that adolescents’ growth curves in the intervention group differ in their inclination. We did not find a correlation between the intercept and slope.

Finally, we used the total sample to test the model displayed in Fig 4. We studied the effect of the intervention, gender, and age on the intercept and slope of prosocial behavior toward strangers. The model showed a good fit: X^2^ = 6.48, df = 4, p = .17, CFI = .99, TLI = .97, SRMR = .03. The results indicated that the Hero program had a significant effect on prosocial behavior toward strangers (*β* = .73, *SE* = .24, *p≤*. 001), while the effects of gender and age were not significant (see Table 3). The R^2^ for the pretest was 54% (*p≤*. 001), that for the posttest was 76% (*p≤*. 001) and that for the follow-up was 67% (*p≤*. 001).

### Prosocial behavior toward friends

**Fig 7:**
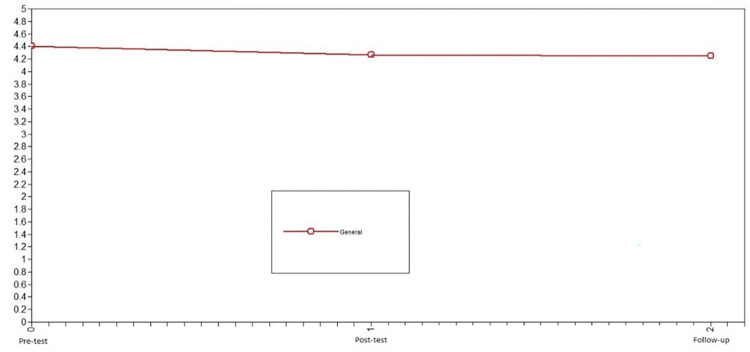
Latent growth curve models of prosocial behavior toward friends of the control group. The best fit in the control group was the model of decreasing linear change.

First, we studied the development of prosocial behavior toward friends in adolescents who did not participate in the intervention (control group) to identify the normative change in the variable. Using LGCMs, we first compared the chi-squared of the no change model vs. linear change model for prosocial behavior toward family. Because this difference was significant, the following step was to compare the linear change model vs. the nonlinear change model; this comparison was not significant. Consequently, the model that presented the best fit in the control group was the linear change model: X^2^ = 8.71, df = 2, CFI = .98, TLI = .97, SRMR = .01 (see comparison in Table 2). For the control group, prosocial behavior toward friends decreased from pretest to follow-up (see Fig 7).

**Fig 8:**
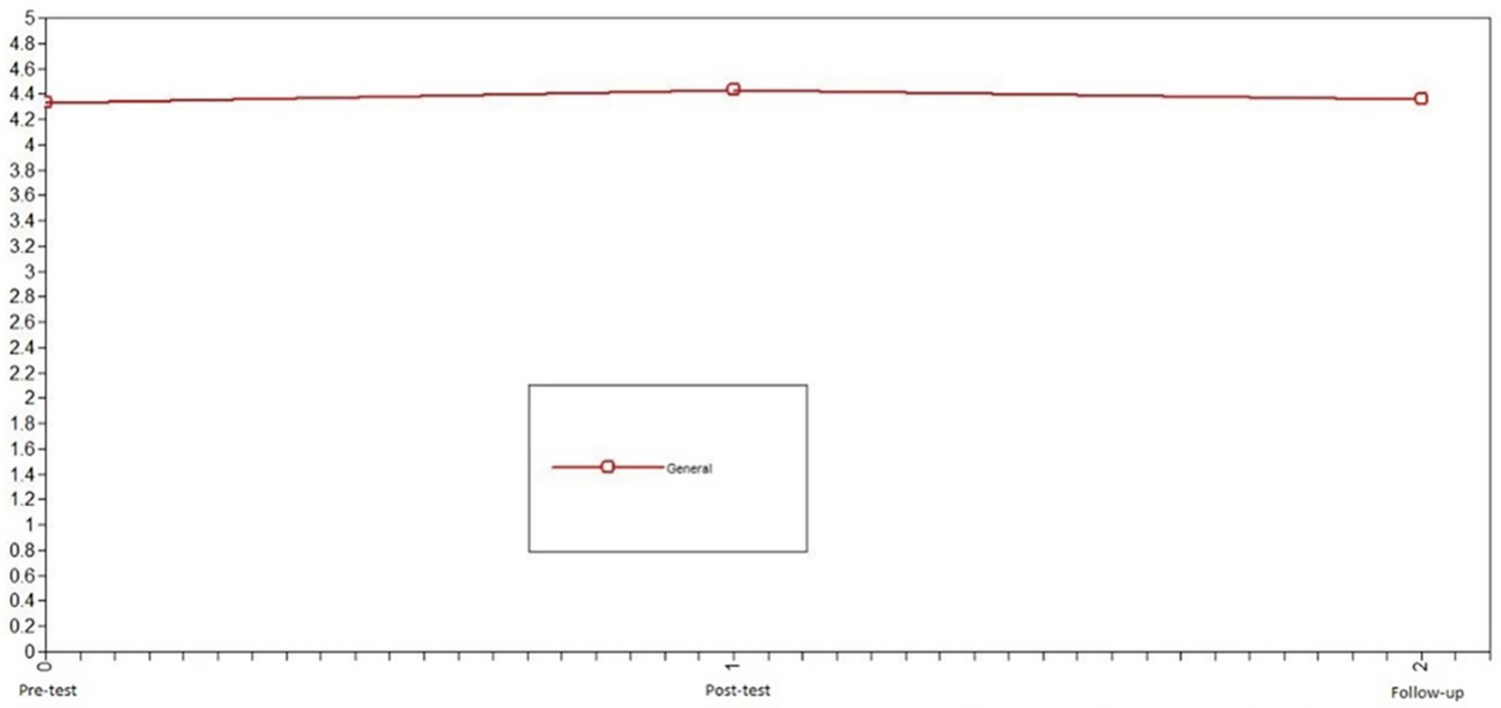
Latent growth curve models of prosocial behavior toward friends of the intervention group. The model that presented the best fit was the nonlinear change model.

Subsequently, we studied the development of prosociality toward friends in adolescents who participated in the intervention to identify the change in the variables promoted by the Hero program. In this case, the developmental trajectory of prosocial behavior toward friends in the intervention group differed over time. The model that presented the best fit was the nonlinear change model: X^2^ = 1.78, df = 1, CFI = .99, TLI = .97, SRMR = .01 (see Table 2). For the intervention group, prosocial behavior toward friends increased from pretest to posttest and remained stable from posttest to follow-up (see Fig 8).

In both the control and intervention groups, we found a significant intercept mean, which indicates individual differences in initial (pretest) prosocial behavior toward friends. Moreover, the variance of the intercept was also significant, which indicates large individual differences in the pretest.

We found a significant negative slope mean in the control group and a nonsignificant slope mean in the intervention group. In the case of the control group, prosocial behavior toward friends decreased over time (S Mean = -.05, *p≤*. 001), while it increased slightly in the intervention group (S Mean = .09, *p =* .05) (see Tables 1 and 2). In addition, because the variance of the slope is significant, we can assume that adolescents’ growth curves in the control and intervention groups differ in terms of slope. A negative correlation between intercept and slope was found in the intervention group (I-S correlation = -.21, *p≤*. 001). Consequently, adolescents with a smaller score on prosocial behavior toward friends at the pretest tended to show a larger slope when the intervention ended.

Finally, we used the total sample to test the model displayed in Fig 4. We considered the effect of the intervention, gender and age on the intercept and slope factors of prosocial behavior toward friends. The model shows a good fit: X^2^ = 0.86, df = 4, p = .93, CFI = 1, TLI = 1, SRMR = .03. The effect of the Hero program on prosocial behavior toward friends’ growth was significant (*β* = .36, *SE* = .18, *p≤*. 001), while the effects of gender and age were not significant (see Table 3). The R^2^ for the pretest was 46% (*p≤*. 001), that for the posttest was 94% (*p≤*. 001) and that for the follow-up was 51% (*p≤*. 001).

### Prosocial behavior toward family members

**Fig 9:**
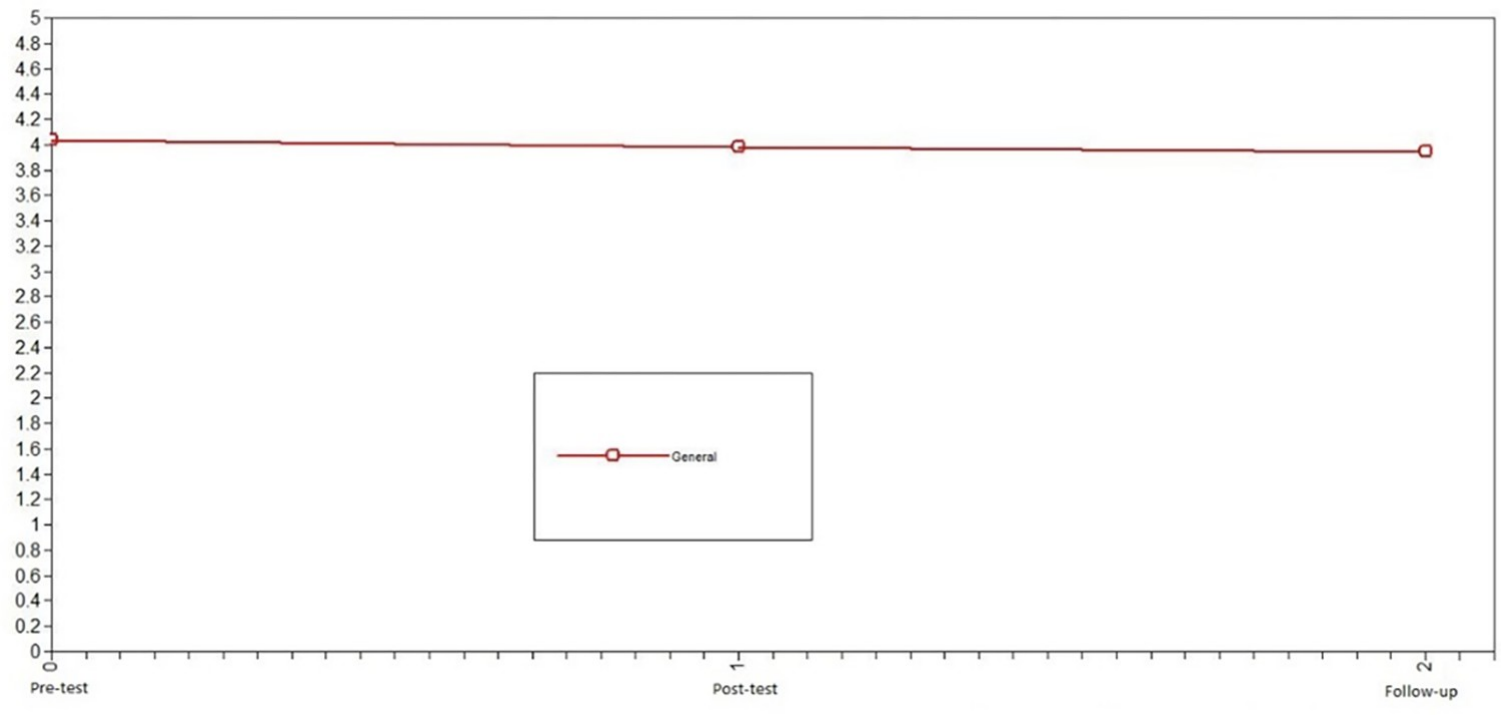
Latent growth curve models of prosocial behavior toward family of the control group. The best fit in the control group was the model of decreasing linear change.

First, we studied the development of prosocial behavior toward family members in adolescents who did not participate in the intervention (control group) to identify the normative change of the variable. Using LGCMs, we first compared the chi-squared of the no change model vs. linear change model for prosocial behavior toward the family. Because this difference was significant, the following step was to compare the linear change model vs. nonlinear change model; this comparison was not significant. Consequently, the model that presented the best fit in the control group was the linear change model: X^2^ = 0.97, df = 1, CFI = 1, TLI = 1, SRMR = .02 (see comparison in Table 2). For the control group, prosocial behavior toward family decreased from pretest to follow-up (see Fig 9).

**Fig 10:**
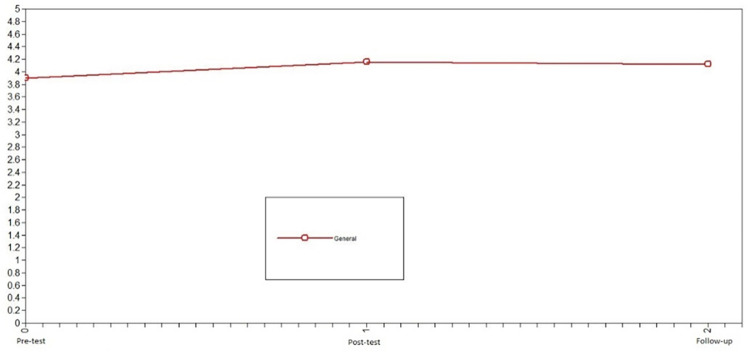
Latent growth curve models of prosocial behavior toward family of the intervention group. The model that presented the best fit was the nonlinear change model.

Subsequently, we studied the development of prosociality toward family members in adolescents who participated in the intervention to identify the change in the variables promoted by the Hero program. In this case, the developmental trajectory of prosocial behavior toward the family differed over time. The model that presented the best fit was the nonlinear change model: X^2^ = 0.24, df = 1, CFI = 1, TLI = 1, SRMR = .02 (see Table 2). For the intervention group, prosocial behavior toward family increased from pretest to posttest and remained stable from posttest to follow-up (see Fig 10).

In both the control and the intervention groups, we found a significant intercept mean, which indicated individual differences in initial (pretest) prosocial behavior toward family. Moreover, the variance of the intercept was also significant, which indicates large individual differences in the pretest.

We found a significant slope mean in the control and intervention groups but with different trajectories. In the case of the control group, prosocial behavior toward family members decreased over time (S Mean = -.03, *p≤*. 05), while that in the intervention group increased over time (S Mean = .24, *p≤*. 001) (see Tables 1 and 2). In addition, because the variance of the slope was significant, adolescents’ growth curves in the intervention group differed in their inclination. A negative correlation between intercept and slope was observed (I-S correlation = -.21, *p≤*. 001): adolescents with a smaller score on prosocial behavior toward family at the pretest tended to show a larger slope factor score when the intervention ended than did adolescents with a larger initial score.

Finally, we used the total sample to test the model displayed in Fig 4. We studied the effect of the intervention, gender, and age on the intercept and slope of prosocial behavior toward family members. The model showed a good fit: X^2^ = 4.40, df = 4, p = .35, CFI = .99, TLI = .99, SRMR = .04. The effect of the Hero program on prosocial behavior toward family was significant (*β* = .54, *SE* = .21, *p≤*. 001), while the effect of gender and age was not significant (see Table 3). The R^2^ for the pretest was 67% (*p≤*. 001), that for the posttest was 84% (*p≤*. 001) and that for the follow-up was 68% (*p≤*. 001).

## Discussion

During 2020, we experienced an exceptional global situation that our generation of adolescents had never experienced before. The measures implemented by the Argentine government to prevent the spread of COVID-19 aimed to avoid physical and social contact, which led adolescents to live situations of strict isolation from their friends, peers and extended family. Magson, Freeman [66] indicated that the principal worry of adolescents during the pandemic was the absence of face-to-face friends and social connections. Moreover, spending a long time interacting with close family (parents and siblings), sometimes in small houses, also increased conflict and discomfort among adolescents [22].

Because adolescents are vulnerable and the pandemic could increase this vulnerability, special consideration from parents, educators, and health professionals is needed. The need to prevent and respond to this imminent discomfort in Argentine adolescents helped us to act quickly to implement a program to support positive emotions and prosocial behavior at the beginning of social isolation. Consequently, three weeks after mandatory confinement for COVID-19 started in Argentina, the Hero program was implemented. The application of this program was an opportunity to help adolescents manage their emotions and instill concern for the needs of others.

Online interventions for adolescents played an important role during mandatory confinement for COVID-19 because they could reach adolescents at home. The Hero program was focused on recognizing one’s own emotions and provided an opportunity to reflect on different positive aspects of life, allowing a change in perspective related to immediate negative events.

The first hypothesis of our study was that adolescents who participated in the home-based Hero program would develop higher levels of positive emotions than those developed by a normative group of adolescents (control group). The results indicated that the normative group was stable with respect to positive emotions throughout the three evaluation periods. We expected to find a decrease in positive emotions because of the social context in which adolescents were living in Argentina and because research conducted with Argentine adolescents indicated that psychological discomfort increased with time [18, 67]. However, we did not find these indicators in our normative sample: the values of positive emotions remained constant over time. This could be explained by the fact that we started the intervention early, during the first three months of mandatory quarantine in Argentina, and the quarantine may not have initially been extremely traumatic or uncomfortable for adolescents. These results are in line with previous studies developed during the pandemic, which also found that positive affect remained stable in adolescence during the first month of lockdown [24]. Moreover, the level of positive emotions in the adolescent intervention group increased through the three evaluation periods, indicating the effectiveness of the Hero program during the pandemic. Thus, the Hero program was effective. Specifically, the intervention group showed an increase in positive emotions in the posttest, and this level remained stable in the follow-up evaluation (three months after the end of the intervention). These results are consistent with previous applications of the Hero program in two Latin American countries prior to the COVID-19 pandemic [51]. In addition, because the variance of the slope was not significant, we assume that adolescents’ growth curves in the intervention group do not differ in inclination. Thus, the growth curves of all adolescents in the intervention group were similar. Finally, for the total sample, we confirmed the effectiveness of the intervention in the promotion of positive emotions, even when controlling for the age and biological sex of the participants.

The Hero program was implemented such that adolescents did not experience the COVID-19 pandemic as passive observers of an “uncontrollable” world situation but rather as an opportunity for personal growth and social involvement. Moreover, because the Hero program was developed to promote prosocial behavior, it provided an opportunity to change adolescents’ perspective from personal worry to concerns about others, including friends, family members, and even strangers in need.

Consequently, the second hypothesis of our study was that adolescents who participated in the home-based program Hero would develop higher levels of prosocial behavior toward strangers, friends and family members than those shown by a normative group of adolescents (control group). The findings indicated that prosocial behavior toward family members and friends decreased in the normative group during the pandemic, while prosocial behavior toward strangers remained stable. These results are in line with a previous study that found no change in prosocial behavior toward unfamiliar others [42, 43] and a decrease in prosocial behavior toward familiar others [42]. It is possible that spending long hours with family members (e.g., parents and siblings) could contribute to generating more conflict, decreasing the motivation to be available to act in a prosocial manner. Several parents worked at home, and they balanced their work obligations with childcare and household chores. These stressful situations likely generated considerable friction in daily interactions, which could cause a decrease in the helping behavior of children. Similar conflicts could emerge among siblings because they share the same common space for a long period of time and sometimes share devices (e.g., telephone, computer) for learning or entertainment. Indeed, a previous study also showed that during the pandemic, time increased family chaos, and family chaos was related to increases in parent–child conflict and sibling conflict and decreased intimacy between family members [68]. There is evidence showing that parent-adolescent relationships worsened during lockdown; in fact, adolescents perceived less parental support and lower positive parenting; in addition, parents and adolescents reported lower levels of warm and supportive parenting [69]. Consequently, it is understandable that poor quality parental-adolescent relationships during the pandemic may lead to a decrease in adolescents’ prosocial behavior toward the family. Concerning friends, interactions between adolescents and their friends were possible only through synchronous classes on virtual platforms such as Zoom and Google Meeting or through virtual video games. The scarce interactions or misunderstandings caused by misuse of virtual communication could have caused a decrease in helping behavior directed toward friends in the normative group. In addition, prosocial behavior toward strangers remained stable, possibly because adolescents naturally did not interact with strangers; that is, adolescents did not intentionally seek the opportunities to help unknown people.

Conversely, the adolescents who participated in the intervention program showed increased prosocial behavior toward strangers, friends, and family members. Notably, the effects of the Hero program were stable over time; that is, the increment remained at the follow-up measurement. Again, the Hero program has been shown to promote prosocial behavior toward different targets, even in the uncertain time of the pandemic [47, 51]. Participation in the program provided an opportunity for adolescents to focus their interests and concerns on others, favoring solidarity in everyday life. In addition, because the variance of the slope was significant in the three models related to prosocial behavior (strangers, friends, and family), we can assume that adolescents’ growth curves in the intervention group differed in inclination. Thus, the growth curve was not similar for all adolescents in the intervention group and needs to be studied in depth.

Finally, when the total sample was studied, we confirmed the effectiveness of the intervention in promoting prosocial behavior toward strangers, friends, and family members, even when controlling for the age and biological sex of the participants. Indeed, when the adolescents finished the Hero program, they commented that it was useful to know when a friend was angry, sad, or worried to understand him or her, be tolerant and help him or her via various activities, such as giving advice to improve relationships with their family or providing help with homework. Participants also felt that the program was helpful with respect to family needs, such as contributing to household chores or looking after younger siblings. Other adolescents reported joining social activities by volunteering to buy necessities for isolated or infected individuals. Furthermore, other adolescents organized a “charity night”, where they walked in groups at night to hand out food to needy people.

## Limitations and future studies

In this study, the main limitation is that 33% of the adolescents included in the intervention group dropped out of the program. Indeed, the intervention group had a higher dropout rate in responses than the waiting list control group (3%). There are three possible explanations for the differences in dropout rate between groups. The first reason may be that the control group was only called to participate in three sessions, while the intervention group was called to participate in eight sessions (7 consecutive sessions and the follow-up evaluation). Especially during the first months of the pandemic, the majority of Argentine families did not have computers or telephones for each member of the family, so parents sometimes could not provide the computer to adolescents during the intervention session. In fact, most of the participants dropped out of the program in the second intervention session. The second reason could be that the control group had the opportunity to participate in the program when the research project was finalized, so they were motivated to participate in the evaluations because they wanted to be included in the program. Third, it is also important to note that the research participants did not receive any payment for participating in the study, which may also have affected adherence. Despite these possible reasons, it is important to highlight that some virtual intervention anticipated a dropout rate of 40% in their studies [70].

Furthermore, self-reports were used to measure the efficacy of the Hero program. In the future, these measurements could be complemented using heteroreports by teachers, parents or peers. In the control and intervention groups, a significant intercept mean was found, which indicates individual differences in initial (pretest) positive emotions and prosocial behavior toward different targets. Consequently, it would be interesting to analyze the trajectories of the different groups of adolescents within interventions and in the normative group. Moreover, it could also be interesting to analyze the LGCMs of different types of positive emotions separately to investigate their similarities and differences.

## Conclusion and future studies

Our findings suggest that the Hero program was a useful online application to improve positive emotions and promote prosocial behavior during uncertain times. The pandemic was accompanied by a series of negative experiences that may have been redefined by the adolescents who participated in Hero. The program developed a virtual space to reflect and train positive socioemotional virtues, which possibly created a positive feedback loop. In other words, the program may promote positive emotions among adolescents, which leads to the development of prosocial behavior toward different targets. In addition, the promotion of prosocial behavior in turn could have promoted positive emotions. Future studies will be necessary to analyze this virtuous circle in the promotion of positive development through the Hero program.

## Supporting information

S1 Data (SAV)
